# Small and Versatile Cyclotides as Anti-infective Agents

**DOI:** 10.1021/acsinfecdis.4c00957

**Published:** 2025-01-22

**Authors:** Elizabete de Souza Cândido, Liryel Silva Gasparetto, Livia Veiga Luchi, João Pedro Farias Pimentel, Marlon Henrique Cardoso, Maria Lígia Rodrigues Macedo, Cesar de la Fuente-Nunez, Octávio Luiz Franco

**Affiliations:** a Programa de Pós-Graduação em Biotecnologia, Universidade Católica Dom Bosco, Campo Grande, Mato Grosso do Sul 79117-900, Brazil; b Programa de Pós-Graduação em Ciências Genômicas e Biotecnologia, Universidade Católica de Brasília, Brasília, Distrito Federal 71966-700, Brazil; c Laboratório de Purificação de Proteínas e suas Funções Biológicas, Universidade Federal de Mato Grosso do Sul, Cidade Universitária, Campo Grande, Mato Grosso do Sul 79070-900, Brazil; d Machine Biology Group, Departments of Psychiatry and Microbiology, Institute for Biomedical Informatics, Institute for Translational Medicine and Therapeutics, Perelman School of Medicine, University of Pennsylvania, Philadelphia, Pennsylvania 19104, United States; e Departments of Bioengineering and Chemical and Biomolecular Engineering, School of Engineering and Applied Science, University of Pennsylvania, Philadelphia, Pennsylvania 19104, United States; f Department of Chemistry, School of Arts and Sciences, University of Pennsylvania, Philadelphia, 19104, United States; g Penn Institute for Computational Science, University of Pennsylvania, Philadelphia, Pennsylvania 19104, United States

**Keywords:** Cyclotides, PAMPs, anti-infectives, multifunctionality

## Abstract

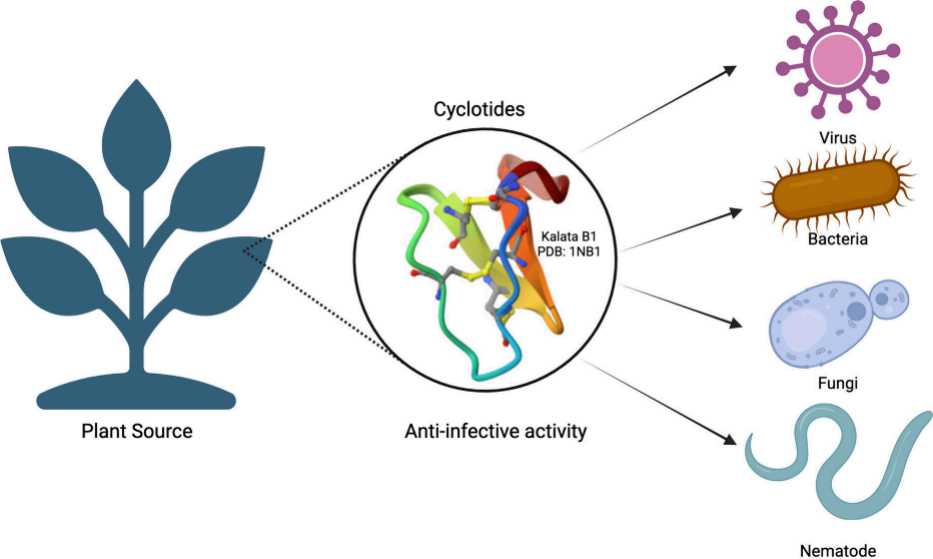

Plants provide an abundant source of potential therapeutic agents, including a diverse array of compounds, such as cyclotides, which are peptides known for their antimicrobial activity. Cyclotides are multifaceted molecules with a wide range of biological activities. Their unique topology forms a head-to-tail cyclic structure reinforced by a cysteine knot, which confers chemical and thermal stability. These molecules can directly target membranes of infectious agents by binding to phosphatidylethanolamine in lipid membranes, leading to membrane permeabilization. Additionally, they function as carriers and cell-penetrating molecules, demonstrating antiviral, antibacterial, antifungal, and nematicidal properties. The structure of cyclotides is also amenable to chemical synthesis, facilitating drug design through residue substitutions or grafting of bioactive epitopes within the cyclotide scaffold to enhance peptide stability. In this review, we explore the multifunctionality of these biomolecules as anti-infective agents, emphasizing their potential as a novel class of antimicrobial drugs.

Throughout evolution, plants have encountered a complex array of abiotic and biotic stresses, leading to the development of sophisticated defense mechanisms to protect against these challenges.^1^ While much has been documented about the anti-infective properties of phytochemicals derived from secondary compounds, less is known about the role of protein compounds in plant defense.

With the increasing drug resistance worldwide, alternative approaches for treating infections are urgently needed. Antimicrobial peptides (AMPs), through their promiscuity, represent excellent candidates.^2,3^ Plants can synthesize AMPs with broad-spectrum activity against bacteria, fungi, and other biotic stresses.^4−7^ Notably, plant AMPs are highly diverse and are classified into multiple families (cyclotides, defensins, thionins, hevein-like peptides, etc.), each with distinct structures and functions.^1,8^

Cyclotides make up one of the most exciting classes of peptides produced by plants. They feature a head-to-tail cyclic backbone composed of approximately 28–37 amino acid residues, complemented by a knotted arrangement of six cysteine residues forming three disulfide bonds, known as a cysteine cyclic knot (CCK). Cyclotides are distinguished by their remarkable stability, including resistance to proteolytic degradation, as well as thermal and chemical stability.^9^

Interestingly, cyclotides can also exist in a linear form, known as acyclotides, which retain the conserved cysteine knot structure and the three disulfide linkages but lack the end-to-end cyclization.^10^ Despite this, their stability remains comparable to that of cyclotides due to the conserved CCK motif.^11^

Cyclotides are typically classified into three subfamilies based on the presence or absence of a *cis*-proline in loop 5. The Möbius subfamily features a twist in the backbone due to the proline residue in loop 5. The bracelet subfamily lacks this proline residue. The third subfamily includes trypsin inhibitors, which possess distinct primary structures compared with the other subfamilies but still maintain the conserved cysteine knot motif (Figure 1A,B). Most acyclotides are also categorized within these three families, characterized by their six loops and the same cystine linkages.^10^ The CCK motif provides stability across all families, offering excellent tolerance to sequence variation.^12,13^

**Figure 1:**
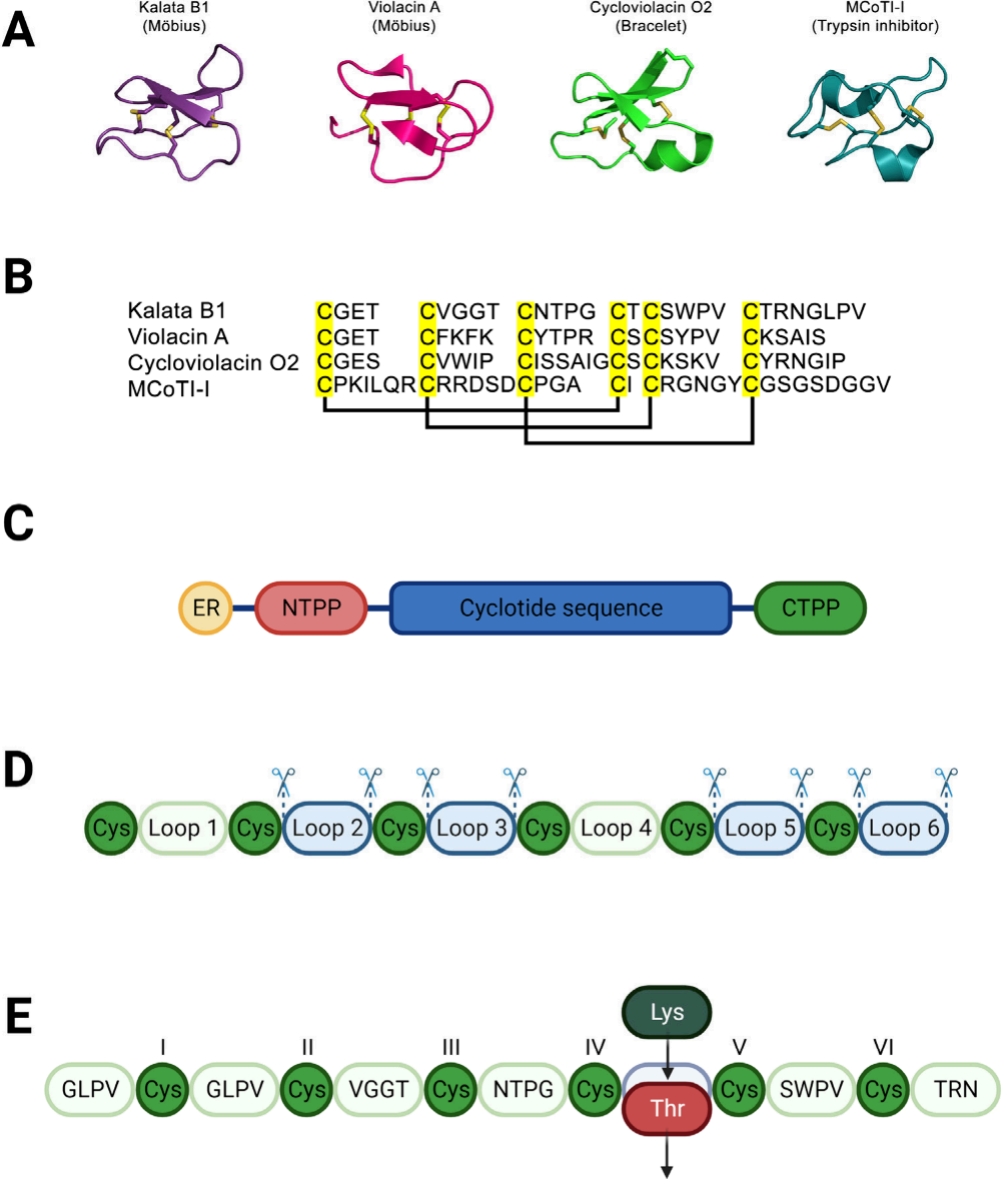
Cyclotides and their flexible scaffold for peptide engineering. (A) Three-dimensional structures of cyclotides: kalata B1 (PDB ID 1NB1) from the Möbius subfamily, cyclotide violacin A (PDB ID 2FQA), also from Möbius subfamily, cycloviolacin O2 (PDB ID 2KNM) representing Bracelet, and the trypsin inhibitor MCoTI-I (PDB ID 5WOW). (B) Representative alignment of the sequences. The disulfide bonds between CysI–CysIV, CysII–CysV, and CysIII–CysVI are highlighted in yellow. (C) Cyclotide precursor protein structure: endoplasmatic reticulum signal sequence (ER, yellow); N-terminal propeptide (NTPP, red); conserved cyclotide sequence (blue); and C-terminal propeptide (CTPP, green). (D) Cyclotide scaffold employed for the recombinant expression of cyclotides. Blue elements symbolize the loops used for grafting with no structural prejudice. (E) Cyclotide scaffold employed for substituting amino acid residues in the sequence. In the example, Thr was replaced by Lys between loops IV and V. Molecules were rendered using the PyMOL Molecular Graphics System, Version 2.5.2 Schrödinger, LLC.

Cyclotides have been isolated from various botanical families, including Violaceae, Rubiaceae, Solanaceae, Cucurbitaceae, Poaceae, and Fabaceae.^14^ According to Cybase (http://www.cybase.org.au), a database dedicated to documenting cyclic proteins,^15,16^ more than a 1,300 cyclotides have been reported as of mid-November 2024. These peptides exhibit a wide range of bioactivities, such as uterotonic,^17^ antimicrobial,^18^ hemolytic,^19^ anti-HIV,^20^ antifouling,^21^ insecticidal,^22^ nematicidal,^23^ and cytotoxic.^24,25^ Some cyclotides also function as protease inhibitors.^26,27^ In plants, cyclotides serve as a defense mechanism against pests, particularly insects and nematodes.^12,28,29^

Cyclotides represent a multifunctional class of AMPs with potential for development as anti-infective agents. While their exact mode of action as anti-infectives is not fully understood, it is believed to involve direct interaction with microbial cell membranes.^30^

Cyclotides are tiny, ultrastable, and multifunctional peptides. They were first discovered by Lorents Gran in indigenous African traditional medicine, specifically in a tea made from *Oldenlandia affinis* (a plant from the Rubiaceae family) used to accelerate childbirth.^17^ The peptide responsible for this activity was later named kalata B1 (kB1), and it was found to have a unique structure and uterotonic activity. After further investigation, this naturally occurring cyclotide was shown to be an AMP (presenting antibacterial, antifungal, anti-HIV and nematicidal activities), leading to the creation of pores, subsequent leakage of cytosolic components from the cell, and, ultimately, cell lysis.^31−33^ The term cyclotide was introduced, in 1999, through the work of Craik and colleagues.^34^

AMPs generally interact with bacteria through electrostatic interactions with the negatively charged bacterial cell membranes (Figure 2).^30^ Similar to linear AMPs, cyclotides can induce bacterial membrane permeabilization (Figure 2B) by disrupting lipid membranes.

**Figure 2:**
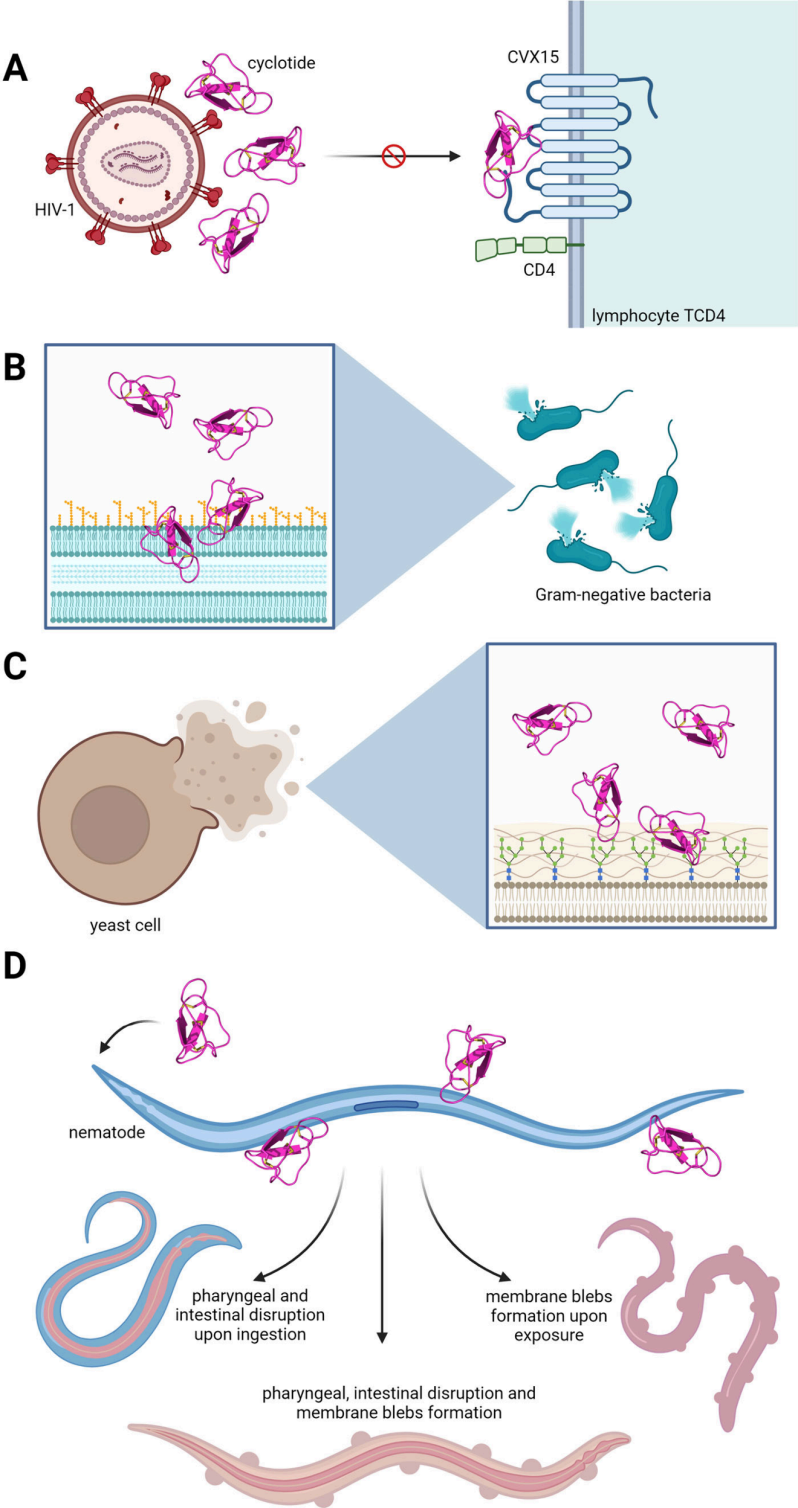
Proposed mechanism of action of cyclotides toward pathogenic organisms. (A) Cyclotides inhibit viral entry by binding to viral receptors, disrupting the viral capsid. Also, cyclotides target the CD4 T lymphocyte receptor molecule CVX15, preventing the viral life cycle from proceeding and reducing HIV replication. (B) Cyclotides act against Gram-negative bacteria by forming pores in the bacterial cell membrane. This pore formation allows ions and molecules to flow, leading to cell death. (C) Cyclotides exhibit antifungal activity by forming pores in the cell membranes of pathogenic fungi. The formation of these pores disrupts cellular homeostasis, resulting in cell death. (D) Cyclotides exert nematicidal activity on young *C. elegans* larvae through ingestion, direct exposure, or both simultaneously. The figure shows cyclotides disrupting the pharynx and intestines of larvae after ingestion, leading to internal damage. Additionally, cyclotides cause membrane blebs on larvae upon exposure, contributing to the nematicidal effect.

Cyclotides have been shown to bind to dodecylphosphocholine (DPC) micelles through a combination of electrostatic and hydrophobic interactions. Furthermore, recent studies postulate that cyclotides may disrupt membranes by forming of multimeric pores, as is the case for kB1.^35^ It has been observed that the lytic effects of cyclotides depend on both their structure and the composition of the lipid membrane.^36^ Moreover, it is known that cholesterol in membranes decreases the activity of all cyclotides and completely prevents membrane lysis through the action of kB1, even at the highest concentrations tested.

The condensing effect of cholesterol on the lipid packing of the membrane results in the continuous organization of the alkyl chain, which leads to increased bilayer thickness. This characteristic is also capable of reducing lateral density oscillations and, thus, bilayer undulations.^36^ These properties, in turn, led to reduced permeability and an increased lateral compressibility modulus, showing an increase in the deformation energy required for entry into the lipid bilayer. As a result, it was observed that cholesterol generally opposes the adsorption and infiltration of peptides into the membrane, which does not necessarily indicate the disruptive potential of the actual adsorbed amount. Furthermore, it is essential to emphasize that the orientation when binding to membranes changes for the cyclotide subfamilies. However, adsorption to the membrane is vital for the lytic activity of the membrane, but the degree of lytic potency and the level of adsorption are not always proportional.

The size and distribution of the hydrophobic surface, net cationic charge, and intramolecular hydrogen bonding potential of the cyclotide are significant factors influencing membrane adsorption and rupture of cyclotides. The extent to which each of these factors contributes is, in turn, driven by the composition of the lipid membrane, such as the presence of cholesterol and the anionic composition. Furthermore, the ability of these small proteins to infiltrate human cell membranes was also verified for MCoTI-II, MCoTI-I, and kB1, allowing them to reach intracellular sites.^37−39^

The factors influencing the bactericidal spectrum of cyclotides can be indicated by the correlation of antimicrobial activity with membrane permeabilization, as already seen in several liposome systems (DPC and phosphatidylethanolamine, PE), in addition to the physicochemical properties of these peptides. Additionally, it has been proven that the chirality of lipid membranes does not interfere in recognizing PE-headgroups, once this process is independent of the phospholipid’s membrane arrangement in d or l. However, the insertion into the bilayer is governed by the chiral environment created by the phospholipids and dependent on the hydrophobic patch.^40^

It is known that general electrostatic and hydrophobic parameters are more important for cyclotides with a broad bactericidal spectrum; on the other hand, a phospholipid-specific mechanism of membrane permeabilization, through interaction with PE lipids, is crucial for cyclotides active mainly against Gram-negative bacteria.^41^

In nematodes, cyclotides exert toxicity without requiring infection; their interaction with the external surface alone is sufficient to affect both adult and larval nematodes (Figure 2D).^42^ The interaction with the nematode’s cuticle, particularly the lipid-rich epicuticle stratum,^42^ suggests that cyclotides may engage with membrane lipid components, leading to membrane disruption. Recent studies have shown that the interaction of certain cyclotides with the nematode *Caenorhabditis elegans* causes damage to the organism’s mouth, pharynx, and midgut through the formation of blebs.^43^

Cyclotides, especially those within the trypsin inhibitory subfamily, are highly effective molecular frameworks for designing AMPs with novel biological activities. These peptides have demonstrated the ability to traverse cellular membranes, but unlike kalata cyclotides, they exhibit no cytotoxicity to mammalian cells at concentrations up to 100 μM.^33,37^ This characteristic enables them to target intracellular protein–protein interactions (PPIs) with minimal side effects on cell viability.^33^

Cycloviolacin O2, used as a template, showed that masking the positive charges caused a reduction in the antimicrobial activity. Based on this assumption, masking the negative charge of the Glu residue was expected to increase activity, but it resulted in almost total loss of antimicrobial activity.^29^ This discovery is supported by the fact that the Glu residue in loop 1 of the cyclotide structure is known to help stabilize the scaffold potentially. Glutamic acid also appears vital for other bioactivities in cyclotides, and masking it distorts the intramolecular interaction rather than the intermolecular interaction with bacterial and other membranes.^13,32^

Studies have shown that the cyclic topology of other cyclic peptides is an essential feature of their anti-infective activity, and opening the scaffold leads to loss of activity.^30,44,45^ Cyclotides also commonly show a cluster of hydrophobic and hydrophilic amino acids exposed on their molecular surfaces, conferring to them an amphipathic nature. This amphipathicity is a common feature shared with conventional AMPs and has been implicated in their antibacterial efficacy.^46^ Hydrophobic amino acid residues also appear to be involved in other biological functions, including anti-HIV activity (Figure 2A). Interestingly, the total net charge does not seem to interfere with anti-HIV activity, but it is known that the more hydrophobic specific loops the peptide has, the more potent this activity will be.^18,32^ Cyclotides prefer certain vesicle sizes, which may explain their selective binding to the HIV capsid over eukaryotic cells.

Cyclotides are active after oral ingestion,^47^ can cross cell membranes,^37^ and effectively target intracellular components *in vivo*.^48^ Along with their stability and tolerance for amino acid substitutions, cyclotides make excellent scaffolds for antibiotic development. While their cyclization does not completely prevent elimination through glomerular filtration, their structural rigidity and lack of terminal residues allow them to overcome the typically short *in vivo* half-life observed for linear peptides, which are prone to degradation by blood serum endo- and exopeptidases.^49,50^

## Anti-infective Activities of Natural Cyclotides

Among circular proteins, cyclotides are the most abundant in plants.^51^ They are ribosomally synthesized molecules composed of an immature protein, which contains an endoplasmic reticulum tag sequence, a pro-region, a highly conserved N-terminal repeat region (NTR), the mature cyclotide, and a C-terminal tail (Figure 1C).^52^ After post-translational modifications, the molecule reaches its mature and cyclized state. Naturally occurring cyclotides present around 28 to 37 amino acids,^53,54^ which can be extended to 45. The enzyme asparaginyl endoproteinase (AEP) is crucial for peptide cyclization in plants. Some studies have demonstrated that a conserved asparagine in cyclotide sequences is essential for post-translational maturation. On the other hand, inactivation of the asparaginyl enzyme leads to a drastic reduction in cyclic products.^52^

Kalata B1 is, by far, the most widely explored cyclotide. Kalata B1 and many other cyclotides have been found in plants commonly used in indigenous or traditional medicines around the world. Table 1 lists native cyclotides with a range of anti-infective activities, such as antibacterial, antifungal, anti-HIV, and nematocidal, along with their peptide subfamily, plant family, and species.

**Table 1:** Native Cyclotides with Anti-infective Activity

cyclotide	family	plant species	botanical family	activity	ref
Cycloviolacin O2	Bracelet	*V. odorata*, *V. tricolor*, *V. baoshanensis*, *V. biflora*, *V. philippica*, *V. uliginosa*, *V. arcuata*, *V. austrosinensis*, *V. anagae*, *Hybanthus enneaspermus*	Violaceae	Antibacterial, Antifungal	(29, 41, 58)
Cycloviolacin O3		*V. odorata*, *V. tricolor*			(41, 58)
Cycloviolacin O8		*V. odorata*		Antifungal	(59)
Cycloviolacin O13		*V. uliginosa*		Antifungal, Anti-HIV	(32, 58)
Cycloviolacin O14	Möbius	*V. odorata*		Anti-HIV	(32)
Cycloviolacin O19	Bracelet	*V. odorata*, *V. tricolor*		Antibacterial, Antifungal	(41, 58)
Cycloviolacin O24	Möbius	*V. odorata*		Anti-HIV	(32)
Cycloviolacin Y1	Bracelet	*V. yedoensis*			(60)
Cycloviolacin Y4					
Cycloviolacin Y5					
Kalata B1	Möbius	*O. affinis*, *V. odorata*, *V. tricolor*, *H. enneaspermus*, *V. baoshanensis*, *V. yedoensis*, *V. philippica*, *V. sumatrana* Miq.	Rubiaceae Violaceae	Antibacterial, Antifungal, Anti-HIV, Anthelminthic	(18, 41, 55, 60)
Kalata B2		*O. affinis*	Rubiaceae	Antibacterial	(41, 61)
Kalata B7				Antifungal	(41)
Kalata B8	Hybrid			Anti-HIV	(62)
Kalata B13	Möbius			Antibacterial, Antifungal	(41)
Varv A/Kalata S		*V. tricolor*	Violaceae		(41, 55, 58)
Circulin A	Bracelet	*Chassalia parvifolia*, *C. chartacea*	Rubiaceae	Antibacterial, Antifungal, Anti-HIV	(18, 20)
Circulin B					
Circulin C					
Circulin D		*C. parvifolia*			
Circulin E				Anti-HIV	(20)
Circulin F					
Vaby A	Möbius	*V. abyssinica*	Violaceae	Antibacterial	(29)
Vaby D					
Cycloviolin A	Bracelet	*Leonia cymosa*		Anti-HIV	(63)
Cycloviolin B					(32, 63)
Cycloviolin C					(63)
Cycloviolin D					
Chassatide C1/C4		*C. chartacea*	Rubiaceae	Antibacterial	(64)
Chassatide C2					
Chassatide C7	Acyclic				
Chassatide C8	Bracelet				
Chassatide C10	Hybrid				
Chassatide C11	Acyclic				
Cliotide T1	Bracelet				
Cliotide T2					(65)
Cliotide T3	Möbius	*C. ternatea*	Fabaceae	Antibacterial	
Cliotide T4					
Cliotide T7	Bracelet				(66)
Cliotide T13 (Cter 23)					
Cliotide T14					
Cliotide T15 (Cter 24)	Möbius				
Cliotide T16	Hybrid				
Cliotide T19 (Cter 26)	Möbius				
Cliotide T20					
Cliotide T21 (Cter 17)	Hybrid				
Cter B	Bracelet			Antibacterial, Antifungal	(41)
Cter E					
					
Cter G					
Cyclopsychotride A		*Psychotria longipes*	Rubiaceae		(18)
Hedyotide B1	Hybrid	*H. biflora*		Antibacterial	(65, 67)
Hedyotide B10					(67)
Hedyotide B11					
Mech 1	Bracelet	*Melicytus chathamicus*	Violaceae		(68)
Mech 2					
Mela 1	Möbius	*M. latifolius*			
Mela 2					
Mela 3					
Mela 4					
Mela 6					
Mela 7					
Panitide L1	Acyclic	*Panicum laxum*	Poaceae	Antibacterial	(69)
Panitide L2					
Panitide L3					
Panitide L4					
Panitide L5	Möbius			Antifungal	
Panitide L6					
Panitide L7					
Panitide L8					
Tricyclon A	Bracelet	*V. tricolor*	Violaceae		(41)
Vila A		*V. labridorica*		Antibacterial	(70)
Vila B					
Vila D	Möbius				
Palicourein	Bracelet	*Palicourea condensata*	Rubiaceae	Anti-HIV	(71, 72)
Varv E/Cylcoviolacin O12	Möbius	*V. yedoensis*	Violaceae		(55, 60)
Vhl-1	Bracelet	*V. hederacea*			(73)
MCoTI-I	Trypsin Inhibitor	*Momordica cochinchinensis*	Cucurbitaceae	Antibacterial	(74)
MCoTI-II					(75)
Gere 1	Bracelet	*Geophila repens*	Rubiaceae	Antibacterial	(76)
Gere 2					
Gere 3					
Gere 4					
Vigno 3, 4, 5, 9		*V. tricolor*	Violaceae	Anti-HIV	(55)
Acyclic vitri E, acyclic cO22, acyclic cO28					
Vitri peptide 2, cO2					
Varv C/D, acyclic vigno 5					
Varv E					
Chacur 1, cO22					

Several plants within the Violaceae family, known to hold cyclotides, are also used as herbs in traditional Eastern medicine. Therefore, bioactivity-guided studies have been conducted on many of them. For example, *Viola tricolor*, a flowering plant from the Violaceae family, is widely used in Russian traditional medicine for its benefits of heat-clearing, detoxification, and alleviating coughs. *V. yedoensis*, found across China, Korea, and Japan, is commonly used as an ingredient of the traditional remedy “Zi Hua Di Ding”, a tea for treating swelling, sores, toxic heat, snake bites, bronchitis, and other diseases. *V. odorata* is also used for treating whooping cough and anti-inflammatory purposes as well as diuretics, expectorants, and other effects. More than 200 cyclotides have been isolated and characterized from this plant family, and some of them have shown antimicrobial and anti-HIV activity.^55−57^

Cyclotides found in the *O*. *affinis* plants have demonstrated anthelmintic activity against gastric parasitic nematodes. Subsequent research revealed that cycloviolacins demonstrated an increase in potency against these nematodes compared to the prototypic cyclotide, kB1.^23^ More studies have indicated that selected cyclotides also negatively affect canine and human hookworms.^77^ Recently, activity of cyclotides was shown toward *C*. *elegans*, showing isolated molecules causing death or damage in the worm’s mouth, pharynx, and midgut. The formation of bubble-like structures known as blebs [sphere-shaped protrusions of the plasma membrane that follow membrane segregation from the main cytoskeleton]^78,79^ around the worm membrane was reported, indicating action through membrane disruption (Figure 2D).^43^

In addition to their anthelmintic properties, cyclotides have shown anti-HIV activity. These studies evaluated a range of prepartions from potentially active fractions rich in cyclotides^55^ to finely characterized molecules. Information is now available on the interaction of kalata B1 with the HIV viral envelope membrane, including mechanistic details^80^ or details on the inhibition of viral replication using the cyclotide modified by grafting MCoTI-I, targeting the CXCR4 cytokine receptor.^78^

Despite many studies demonstrating anti-infective activity, concerns remain regarding these molecules’ cytotoxicity and hemolytic potential, which limit their further development.^80^ Notwithstanding their impressive immunomodulatory capacity, it is important to note that some cyclotides may have toxic characteristics to human cells. The researchers point out that the cytotoxicity of cyclotides is concentration-dependent and can be highly affected by substitutions or removal of amino acids.^81,82^ That said, we emphasize that the preference of cyclotides for cancer cell membranes over healthy cells has been reported.^37,40,80^ As mentioned previously, it is believed that cyclotides may target phosphatidylethanolamine (PE)-rich phospholipids and thus enter the cell through endocytosis and membrane translocation.^40^

## Cyclotides as Versatile Anti-infective Agents

Due to their CCK topology, cyclotides exhibit a high tolerance for sequence variability and mutations. This characteristic makes them ideal candidates for biotechnological applications that exploit their resistance to physicochemical and biological degradation.^79,83^ Additionally, cyclotides can be modified to optimize their activity and explore their therapeutic potential through precise amino acid alterations or the insertion of an amino acid sequence in one of their backbone loops.^84,85^

One promising strategy is grafting, which was initially introduced in horticulture by merging a scion from one plant with the rootstock of another to create a new plant with combined properties.^86^ This method can also be applied at the molecular level, allowing for the intentional combination of two molecules for therapeutic purposes.^87,88^ Grafting linear bioactive peptides, known as epitopes, into the structural framework of cyclotides presents a promising strategy against infectious diseases, harnessing the therapeutic benefits of both peptide sequences.^75^

Grafting is a valuable pharmacological alternative, since many bioactive peptides are highly susceptible to proteolytic degradation when administered to the human body. Hence, grafting these peptides onto a cyclotide structure can increase their bioavailability and promote their penetration into target cells, enhancing the treatment efficacy.^50,89,90^ Additionally, the cyclotide structure remains as the CCK motif can house an extensive series of loop sequences, potentially enabling the incorporation of pharmaceutical epitopes. Such adaptability holds promise for developing innovative anti-infective drugs, maintaining the overall fold and CCK stability.^91^ However, among the six loops of a cyclotide, loops 1 and 4 play crucial roles in preserving the disulfide bonds and normally cannot be used for grafting bioactive peptides. Consequently, only specific segments of loops 2, 3, 5, or 6 can be substituted with the epitopes, ensuring that the stability of the CCK framework remains intact (Figure 1D).^87^

Although many peptides have already been used for grafting within cyclotides, particularly in the context of diseases such as cancer^88^ and multiple sclerosis,^92^ the grafting of antimicrobial peptides for infectious disease management is still limited. Despite their versatility, cyclotide synthesis remains a major bottleneck and limits their large-scale use. Plants ribosomally synthesize cyclotides, but they can be produced through various chemical strategies. They are predominantly synthesized by using Fmoc-based SPPS (solid phase peptide synthesis) approaches. However, access to grafted peptides by stepwise SPPS can be inefficient, leading to low yields of crude peptides. Furthermore, oxidative folding of grafted peptides is often highly compromised due to the inserted sequence, which causes structural disruption that does not allow formation of the native CCK scaffold. To circumvent this situation, a modular “plug and play” synthesis approach was recently reported.^93^ The authors describe the addition of bioactive epitopes to an already folded scaffold peptide. This strategy allows for the reliable insertion of structurally diverse epitopes and, consequently, the rapid diversification of the scaffold. In this way two bottlenecks of cyclotide synthesis are overcome: first, the stepwise assembly of longer and more difficult sequences and, more importantly, the frequently substantially impaired folding of grafted peptides.

Antimicrobial peptides grafted into a cyclotide are more likely to reach their bacterial targets and effectively bind the extracellular receptors associated with biofilm formation and microbial growth.^51,76^ The use of this technique to combat pathogenic microorganisms was recently explored in a clever comparison of a 9mer AMP in its linear form, cyclized and grafted into loops 5 and 6 of MCoTI-II (which does not present prior antimicrobial activity) against *Acinetobacter baumannii*, *Staphylococcus aureus*, *Klebsiella pneumoniae*, *Escherichia coli*, *Pseudomonas aeruginosa*, and *Enterococcus faecalis*. This study demonstrated that the cyclization and grafting processes provided excellent stability to the molecules in the culture medium and human serum. Nevertheless, the linear molecule continued to be more effective. The three tested forms of the peptide demonstrated low cytotoxicity and hemolytic potential.^75^

Moreover, cyclotides, such as circulins, cyclophilins, and palicourein, have already been described to display antiviral activity, especially against enveloped viruses, such as HIV, in very low concentrations.^80^ It has been reported that the anti-HIV activity of kalata B1 is due to its ability to bind and disrupt viral capsid membranes rich in phosphatidylethanolamine phospholipids.^78^ Cyclotides can thus interfere with interactions between viruses and the cell by preventing interaction with extracellular receptors. This can be achieved through the cyclotide’s interaction with a viral co-receptor on the target cell, thereby blocking its binding site and preventing the virus from entering host cells.^94^

For intracellular targets, cyclotides can penetrate cell membranes by mechanisms like macropinocytosis and various endocytic pathways facilitated by their interaction with phosphatidylethanolamine (POPE) and the modulation of membrane curvature^95,96^ and *in silico* simulations demonstrated that charged cyO2 residues interact with POPE amino and phosphate head groups more strongly. On the other hand, hydrophobic residues of this molecule appear deeply inserted into the lipid layer of the membrane, thus forming bound complexes. Conversely, phosphatidylcholine (POPC) lipids with three methyl groups in the amino headgroup create a barrier when interacting with cyO2. As a result, relatively more challenging binding of cyO2 is observed in POPC compared to POPE.^95^ Hence, grafted cyclotides present a versatile approach to combat infectious agents through multiple antimicrobial mechanisms.

For instance, a novel antibacterial cyclotide with broad-spectrum activity was developed by grafting into loop 6 of the MCoTI-I cyclotide framework a modified PG-1 sequence. This engineered cyclotide exhibited remarkable stability, maintaining the native cyclotide conformation and enabling the epitope to assume its natural bioactivity. Furthermore, it demonstrated low toxicity and *in vivo* efficacy, showing high potential as a therapeutic candidate for treating bacterial infections.^74^ Moreover, this approach can generate drugs against other infectious pathogens including viruses. Cyclotides can target the viral envelope precisely through the viral lipid envelope interaction, which leads to pore formation and leakage of internal particles.^55,97,98^ Therefore, researchers engineered MCo-CVX-5c, a cyclotide variant formed by grafting the polypeptide CVX15 polypeptide into loop 6 of MCoTI-I. This modified cyclotide effectively suppresses HIV-1 replication by targeting the critical CXCR4 receptor, preventing HIV-1 entry into human lymphocytes.^78,87^ Cyclotides have been described to cause membrane disruption, leading to antiviral activity.^99−101^

## Can Cyclotides Be Developed as Novel Anti-infectives?

Cyclotides are well-studied molecules with detailed structural, functional, and genetic characterization. These microproteins have been widely explored for their bioactivities and potential as biotechnological tools. With over 50,000 predicted cyclotides, many untested cyclotides or engineered analogs could be even more powerful than those currently described, emphasizing the need to further investigate their antiviral potential. Despite the desirable features of cyclotides, such as their unique scaffold, capacity to infiltrate cells, and exceptional thermal and chemical resistance, their application as anti-infectives has not been fully explored. However, early findings suggest that cyclotides can be safely administered orally, as demonstrated by the T20K molecule.^102^ However, this research aims to treat diseases such as multiple sclerosis or cancer.^92,103,104^ Nevertheless, it is extremely important to emphasize that there are still few studies that address the oral bioavailability of cyclotides,^105−107^ which leads us to the need for further studies before these molecules become commercially available drugs.

At this point, it is feasible to consider the applicability of these innovative techniques to the emergence of new anti-infective drugs. Gaining insights into the enzymatic cyclization mechanism within cyclotide biosynthesis is significant for advancing *in vitro* cyclization techniques (Figure 1D). This advancement, in turn, streamlines the scaling of processes for pharmaceutical and agricultural applications on a broader scale.^108^

Some alternatives are used to achieve post-translational modifications of cyclotides *in vitro*. Approaches encompass the utilization of thioester-mediated cyclization facilitated by modified inteins. Additionally, thiol-induced selective N→S acyl-transfer is implemented, along with the application of intein-mediated protein trans-splicing (PTS).^109^ Inteins find widespread application in the *in vitro* production of peptides; it would be no different for cyclotides. Numerous studies employing recombinant expression have demonstrated that these frameworks prove invaluable for researchers striving to achieve cyclization of cyclotides within a laboratory environment.^110,111^

Sustainable and environmentally friendly techniques are also being explored to produce engineered cyclotides. Using transgenic plants showed a promising and economically favorable alternative for this purpose. Thus, T20K kalata B1’s example successfully expressed this cyclic peptide in *Nicotiana benthamiana*.^102,104^ Kalata B1 is a naturally occurring cyclotide and showed complete resistance to degradation in gastric and intestinal digestion models, making it a strong candidate for oral administration (Figure 1E).

Still, the use of cyclotides in the formation of nanotubes was recently presented, reinforcing their versatility as a molecule. The study shows the cyclotides as an effective, biocompatible, and stable delivery system. The cyclotides are self-assembled through peptide aggregation into a fairly stable conformation of nanotubes, thanks to the core full of cysteine knots, which force the hydrophobic residues to be exposed on the structure surface. It was proven that cyclotide nanotubes remain stable when subjected to temperatures of up to 200 °C and have a durability of up to 3 months when stored under refrigeration at −20 °C.^112^

Even considering the use of cyclotides as a delivery system, these molecules have been widely explored as cell-penetrating peptides (CPPs). However, there is a genuine concern for making the internalization of these molecules more effective. Studies with MCoTI-II^113^ have shown encouraging results with strategies involving the balance of positive and negative charges on the molecule surface, ensuring its structural stability and entry into the cell, combined with grafting of the desired peptide fragment. Thus, using the cyclotide scaffolds as carriers of biomolecules into cells is a more attractive alternative than linear CPPs.^113^

## Conclusions and Perspectives

As the urgent need for new anti-infective agents continues to grow, ultrastable and versatile molecules like cyclotides represent excellent candidates. These molecules offer promising pharmaceutical potential, as seen in their application in treating multiple sclerosis and various cancers. However, transitioning from academic studies, which have demonstrated their effectiveness, to preclinical and clinical studies is necessary. The T20K peptide is a significant driver in this regard, as it is already in preclinical studies and is moving toward phase I clinical studies.^102,104^ Even so, it is imperative to remember that the use of medicines or food additives derived from cyclotides or any other peptide, must comply with the local legislation of each country. There are no recommendations regarding the use of cyclotide-based drugs described by the European Medicines Agency (EMA) or the Food and Drug Administration (FDA). However, the FDA has a section that guides all peptide-based drugs, section 505(b) of the Federal Food, Drug, and Cosmetic Act (FD&C Act), which details all clinical pharmacology considerations, drug–drug interactions, prolongation and immunogenicity risk on a peptide drug product’s pharmacokinetics, safety, and efficacy. Therefore, it is crucial that new drugs or dietary supplements that are developed carefully comply with these recommendations to ensure the safety of patients/consumers. While there is still much to explore, especially in controlling infectious diseases caused by bacteria and fungi, the application of artificial intelligence (AI) could significantly accelerate the design and discovery of peptides, leading to the development of novel anti-infectives and other therapeutic molecules.^114−116^ Despite the challenges, significant progress has already been made in combating viral and parasitic infections using cyclotide-based drugs. The inherent flexibility of cyclotides for optimization offers a unique opportunity to further develop these agents as therapeutics. While cyclotides represent a promising avenue for drug development, substantial work remains to ensure the safe and effective use of cyclotide-based anti-infectives in clinical settings.

## Abbreviations

AEP: asparaginyl endoproteinase
AMP: antimicrobial peptide
CCK: cysteine cyclic knot
CyO2: cycloviolacin O2
KB1: kalata B1
NTR: N-terminal repeat region
PG1: protegrin 1
POPC: phosphatidylcholine
POPE: phosphatidylethanolamine
PTS: protein trans-splicing
